# Development and Validation of a Novel Platform-Independent Metastasis Signature in Human Breast Cancer

**DOI:** 10.1371/journal.pone.0126631

**Published:** 2015-05-14

**Authors:** Shuang G. Zhao, Mark Shilkrut, Corey Speers, Meilan Liu, Kari Wilder-Romans, Theodore S. Lawrence, Lori J. Pierce, Felix Y. Feng

**Affiliations:** 1 Department of Radiation Oncology, University of Michigan Medical School, Ann Arbor, Michigan, United States of America; 2 Comprehensive Cancer Center, University of Michigan Medical School, Ann Arbor, Michigan, United States of America; 3 Michigan Center for Translational Pathology, University of Michigan Medical School, Ann Arbor, Michigan, United States of America; Cedars Sinai Medical Center, UNITED STATES

## Abstract

**Purpose:**

The molecular drivers of metastasis in breast cancer are not well understood. Therefore, we sought to identify the biological processes underlying distant progression and define a prognostic signature for metastatic potential in breast cancer.

**Experimental design:**

*In vivo* screening for metastases was performed using Chick Chorioallantoic Membrane assays in 21 preclinical breast cancer models. Expressed genes associated with metastatic potential were identified using high-throughput analysis. Correlations with biological function were determined using the Database for Annotation, Visualization and Integrated Discovery.

**Results:**

We identified a broad range of metastatic potential that was independent of intrinsic breast cancer subtypes. 146 genes were significantly associated with metastasis progression and were linked to cancer-related biological functions, including cell migration/adhesion, Jak-STAT, TGF-beta, and Wnt signaling. These genes were used to develop a platform-independent gene expression signature (M-Sig), which was trained and subsequently validated on 5 independent cohorts totaling nearly 1800 breast cancer patients with all p-values < 0.005 and hazard ratios ranging from approximately 2.5 to 3. On multivariate analysis accounting for standard clinicopathologic prognostic variables, M-Sig remained the strongest prognostic factor for metastatic progression, with p-values < 0.001 and hazard ratios > 2 in three different cohorts.

**Conclusion:**

M-Sig is strongly prognostic for metastatic progression, and may provide clinical utility in combination with treatment prediction tools to better guide patient care. In addition, the platform-independent nature of the signature makes it an excellent research tool as it can be directly applied onto existing, and future, datasets.

## Introduction

The prognostic classification of breast cancer has historically been based on clinical and pathologic variables such as endocrine receptor status, patient age, histological grade, and stage [1], with molecular subtypes now beginning to supplant endocrine receptor status [2–4]. More recently, analysis of gene expression has greatly improved prognostic ability and led to the adoption of commercially available gene signatures which include MammaPrint (Agendia) and Oncotype Dx (Genomic Health Inc). These clinical and pathologic risk stratifiers and commercially available gene signatures have all been based on clinical performance as an endpoint and therefore incorporate some combination of the intrinsic metastatic potential of the tumor and its resistance to conventional treatments [5, 6]. Prosigna (Integrated Oncology) is another commercial prognostic signature based on the PAM50 intrinsic molecular subtyping of breast cancer, which was also not developed to predict intrinsic metastatic potential [7]. Thus, current molecular diagnostics cannot determine why subsets of patients do poorly and whether this is related to a tumor’s ability to metastasize at baseline or the inherent resistance to treatment such as chemotherapy, endocrine therapy, or radiation. A signature to predict the metastatic potential of a tumor could be clinically useful in conjunction with more specific treatment-resistance signatures and allow for identification of the underlying factors governing poor outcomes in patients, thus guiding personalized treatment.

To develop a signature for intrinsic metastatic potential of breast cancer, the starting point must be an *in vitro* or *in vivo* model system, since cohorts of untreated breast cancer do not exist. Metastasis is a multi-step process in which tumor cells invade locally, intravasate into a blood vessel, survive in the bloodstream and stop at a distant organ site, then extravasate, survive, and colonize that site [8, 9]. A common *in vitro* assay to assess invasion is the Boyden chamber assay, which Neve *et al*. utilized to report metastatic potential in a large collection of breast cancer cell lines [10]. However, invasion is just one of the required steps for metastasis, and *in vitro* assays have difficulty capturing all steps in a single experiment. *In vivo* model systems such as xenografts in immunodeficient mice represent an alternative where metastasis can be observed in the whole organism. This approach has been used to characterize metastatic potential for a handful of breast cancer cell lines [11, 12], and a xenograft approach with a small number of cell lines has been used to develop a breast cancer lung-metastasis signature [13]. However, no studies report large scale results, likely due to the technical challenges of this system [14]. An attractive model system which balances efficiency while still encapsulating all steps of metastasis *in vivo* is the Chick Chorioallantoic Membrane (CAM) assay, where both micro- and macro- metastases from tumor cells placed on the chorioallantoic membrane of a chick embryo can be quantified in end organs [15, 16].

Using this system, we report the first high-throughput analysis of gene expression data from an *in vivo* metastasis screen in breast cancer. We hypothesized that by pairing metastatic potential, as assessed by the *in vivo* CAM assay, with gene expression profiles from 21 preclinical breast cancer models, we would be able to develop a signature to predict the intrinsic metastatic potential of breast cancer. We then trained and cross-validated our results in 327 breast cancer patients and subsequently validated this metastasis signature (M-Sig) on four independent clinical breast cancer datasets with 1467 women who were profiled on different microarray and RNAseq platforms and who had undergone a wide range of treatments. We demonstrate that our signature accurately and consistently identifies patients likely to develop metastasis independent of the method of obtaining tissue, the platform of gene expression profiling, and treatment. This is the first study to identify and validate a signature of intrinsic metastatic potential based on a large scale *in vivo* model system screen and may aid in elucidating the biological mechanisms of metastasis in breast cancer.

## Materials and Methods

### Ethics statement

The University of Michigan’s Committee on the Use and Care of Animals (UCUCA) granted a waiver to perform the embryo experiments as the embryos used in this study were all in early stages of embryonic development and were used before day 21 when the embryo is viable, and thus committee review and approval was not necessary.

### Cell culture and cell lines

Breast cancer cells were propagated from frozen samples in cell culture media, and passaged when reaching confluence. Cell lines were chosen to include an appropriate representation of all molecular subtypes. ACC cell lines were purchased from the Deutsche Sammlung von Mikroorganismens und Zellkulturen GmbH (DSMZ, Brunswick, Germany) while the remaining cell lines were purchased from ATCC. All cell lines were purchased between 07/2012 and 01/2014. All cell lines were characterized and genotyped immediately prior to evaluation at the University of Michigan DNA Sequencing core facility by fragment analysis and ProfilerID utilizing the AmpFLSTR Identifier Plus PCR Kit (Life Technologies, Grand Island, NY, Cat #4322288) run on an Applied Biosystems AB 3730XL 96-capillary DNA analyzer. Sample fragments were compared against cell line standards provided by ATCC and DSMZ. ZR75-30, MDA-MB-231, MDA-MB-453, BT474, BT20, AU565, HCC 1954, HCC 1806, HCC38, HCC70, and HCC 1937 breast cancer cell lines were grown in RPMI 1640 (Invitrogen, Carlsbad, CA) supplemented with 10% FBS (Invitrogen) in a 5% CO2 cell culture incubator. ACC-231 cells were grown in 90% RPMI medium (Invitrogen) supplemented with 10% FBS (Invitrogen) in a 5% CO2 cell culture incubator. ACC-302 cells were grown in 80% DMEM (Invitrogen) supplemented with 20% FBS (Invitrogen) in a 5% CO2 cell culture incubator. ACC-422 cells were grown in 85% MEM (Invitrogen) supplemented with 15% FBS (Invitrogen) in a 5% CO2 cell culture incubator. MDA-MB-361, BT549 and T47D cells were grown in RPMI 1640 (Invitrogen) supplemented with 10% FBS (Invitrogen) and 0.023 IU/ml insulin in a 5% CO2 cell culture incubator. ACC-459, ACC-440, CAMA-1, were grown in DMEM (Invitrogen) supplemented with 10% FBS in a 5% CO2 cell culture incubator. MCF-7 cells were grown in modified MEM (Invitrogen) with 0.023 IU/ml insulin in a 5% CO2 cell culture incubator. All cultures were maintained with 50 units/ml of penicillin/streptomycin (Invitrogen).

### CAM assays

CAM assays were performed as previously described [17]. Briefly, fertilized eggs were purchased from Charles River Laboratories (Franklin, CT) and were incubated at 37°C for 10 days in a rotary incubator. At day 10, a small window was made in the shell above the dropped CAM. Cultured cells were detached by brief trypsinization and washed in PBS. Two million cells were re-suspended in a solution composed of 75% of corresponding but serum-free tissue culture media and 25% Matrigel (BD Biosciences, San Jose, CA) and were placed over the CAM. Following inoculation, the windows were sealed and the eggs were returned to a stationary incubator for seven days. On day 17, the embryos were removed from the eggs and the liver and both lungs were harvested, washed in cold PBS and were kept at -80°C until day of DNA extraction.

### DNA extraction and qPCR for Alu repeats

The number of human breast cancer cells within chicken embryo livers and lungs was determined by quantitative polymerase chain reaction (qPCR) for human Alu repeats in total DNA obtained from liver and lungs of chicken embryos [18]. Liver and lung DNA was extracted with DNeasy Blood and Tissue kit (Qiagen, Venlo, Netherlands). Livers and lungs were thawed, washed with PBS, homogenized in cell lysis buffer (5 mL for liver, 3 mL for lungs), and incubated overnight with proteinase K (Qiagen) at 55°C and after that with RNase (Qiagen) at 37°C for 30 min. After protein precipitation, DNA was extracted by adding isopropanol and centrifugation. The DNA pellet was re-suspended in DNA hydration solution and diluted up to 0.2 micrograms/microliter. Human Alu sequences were amplified by qPCR from 0.2 ug/uL genomic DNA in a 20 uL reaction with 1 uL probe solution that was prepared from 30 uL Alu-sense (5′-GTCAGGAGATCGAGACCATCCT-3′) and 30 uL Alu-antisense (5′-AGTGGCGCAATCTCGGC-3′) primers, and 30 uL TaqMan probe (5′-6-FAM-AGCTACTCGGGAGGCTGAGGCAGGA-TAMRA-3′). qPCR assays were carried out in triplicates and included DNA from chick embryos with a sham operation as a negative control. DNA for the standard curve was obtained from corresponding breast cancer cell lines by serial dilutions to 0, 0.01, 0.1, 1, 10, 1000, and 10000 cells in the sample. The estimated cell count for lung and liver metastases was calculated, and the difference was taken from an equal number of controls. The metastasis score was calculated by taking the sum of these normalized lung and liver cell counts at 7 days post-application for all 21 cell lines.

### Feature selection

Normalized gene expression data for 20 of the 21 cell lines were obtained from Hoeflich *et al*. [19] (microarray data for AUA565 was unavailable). We used Pearson’s correlation to compare the log CAM metastasis score to the log expression of every gene on the cell line microarrays. Fold change for each gene was calculated by taking the difference between the 25th and 75th percentiles of expression. Only genes with high correlation (Pearson’s correlation p < 0.05), a wide range of expression (fold change > 2), high expression (median log_2_ expression > 2), and present on all microarrays from the clinical training and validation cohorts were retained and used in the final model. Pathway analysis was performed on this set of genes using the Database for Annotation, Visualization and Integrated Discovery (DAVID) using the following databases: Gene Ontology (GO) Biological Processes, Reactome, KEGG, Biocarta, and Panther pathways [20, 21]. Processes and pathways were then manually curated to exclude terms that were too broad, or were duplicated in prior terms.

### Training and validation

We used five publicly available breast cancer clinical cohorts and their associated clinical and normalized gene expression data as described previously. Analyses were designed in accordance with REMARK criteria when possible and appropriate [22]. We divided these cohorts into a training/cross validation cohort (Kao *et al*. [23]), and four validation cohorts (Wang *et al*. [24], van de Vijver *et al*. [25], Hatzis *et al*. [26], and The Cancer Genome Atlas (TCGA) [4] with normalization described in [27]). The MIAME compliant datasets were downloaded from the Gene Expression Omnibus (GEO) database with series number GSE20685 (Kao), GSE2034 (Wang), and GSE25066 (Hatzis). Data for the van de Vijver cohort was obtained from http://microarray-pubs.stanford.edu/wound_NKI/explore.html (also available from http://www.oncomine.org), and data for the TCGA cohort was downloaded from http://tcga-data.nci.nih.gov. Expression levels were log transformed, median centered and scaled. The complete set of genes selected from the cell lines described above was used to train a Random Forest model with 100,000 trees in the training cohort [28]. This model (M-Sig) produced a score ranging from 0 to 1 which represents the proportion of trees in the Random Forest which predict that a particular patient will metastasize. This M-Sig score is the model’s estimate of the risk of metastasis. Performance in the training set was evaluated using the model predictions as well as the out of bag (OOB) error rate, an internal cross-validation mechanism of Random Forest models. This signature was then used to classify the validation cohort into high and low risk for metastasis groups using an M-Sig score cutoff of 0.5.

### Statistical analysis of clinical outcomes

In order to log transform the metastasis scores for plotting, one was added to all scores prior to log transformation such that a metastasis score of 0 is 0 after transformation. The performance of the Random Forest predictions with clinical outcomes was performed using the Log-rank test comparing the predicted high versus low risk groups in each of the clinical cohorts, and Kaplan-Meier curves were generated. The Cox proportional hazards model was used for all hazard ratios and confidence intervals as well as for univariate and multivariate analysis in cohorts with additional clinical and pathologic data. Covariates in the multivariate models were limited to what was publicly available, and were used as continuous variables unless otherwise noted. A prediction curve was generated by fitting a logistic regression model to the OOB prediction versus the outcome data in the training cohort. The predicted curve and 95% confidence interval was subsequently plotted. All models and statistics were performed using R [29].

## Results

### Assessment of metastases in an in vivo model

**Fig 1:**
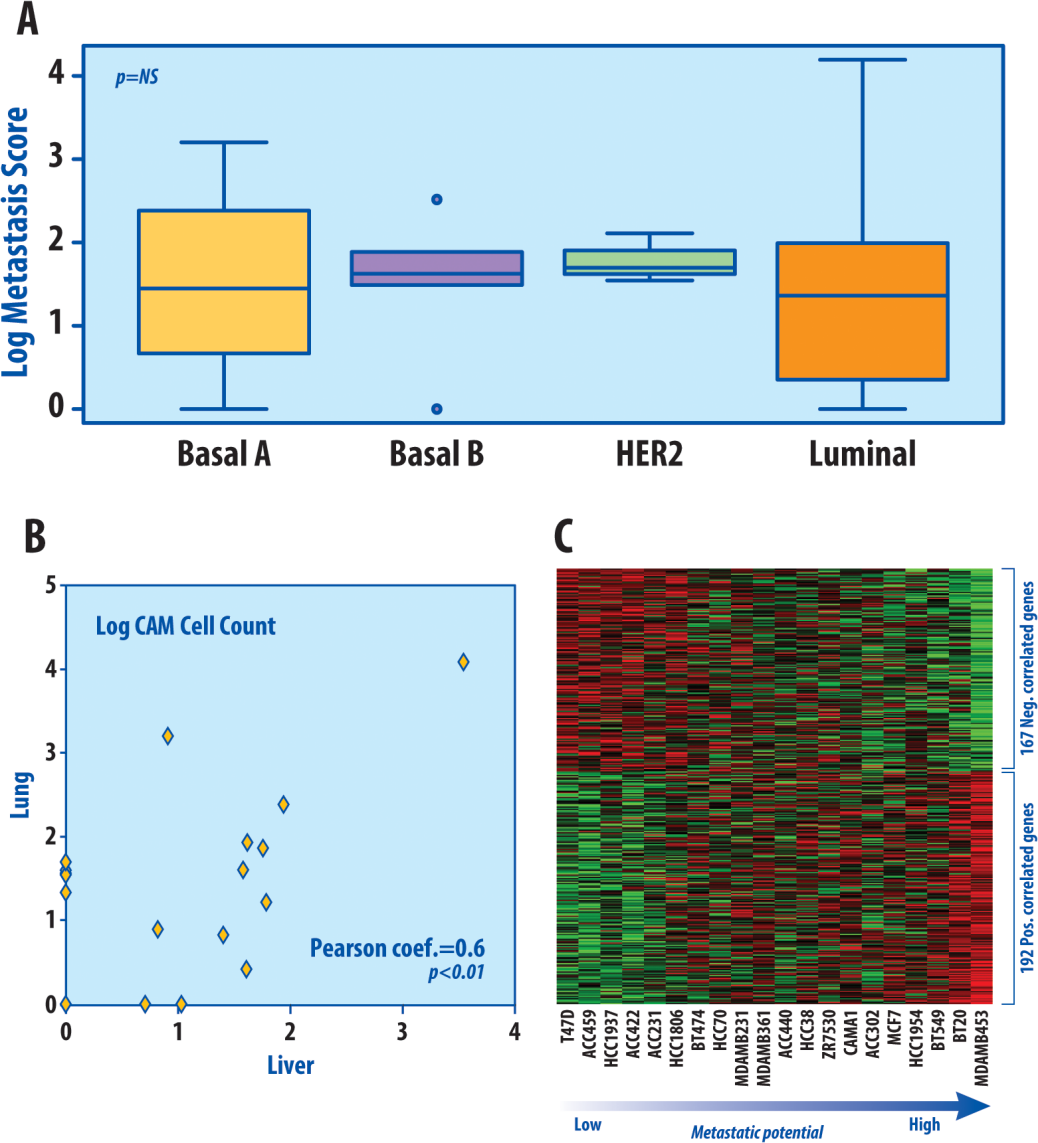
CAM assays. (A) Box plot depicts the metastasis scores of the cell lines grouped by molecular subtype. Metastasis signature scores across breast cancer cell lines were not significantly associated with molecular subtype using ANOVA on the original non-log scores. (B) Scatter plot depicting the log CAM cell counts in the lung and liver, which were highly correlated (Pearson’s correlation coefficient = 0.6, P-value <0.01). (C) Heatmap showing the expression of the genes that were most highly correlated with CAM metastasis score in breast cancer cell that also had a minimum level of expression and internal variability. The genes are ordered by correlation coefficient and the cell lines are ordered by increasing metastatic potential and metastasis score.

We first sought to determine the intrinsic metastatic potential of 21 breast cancer cell lines using the *in vivo* CAM assay model system. Using this system, metastatic cell counts in the chick embryo liver and lung were quantified, and the sum of the two was used as a metastasis score (MS). Table 1 shows the CAM quantification in the chick embryo lung, liver, and the normalized sum of the two (the metastasis score). CAM assay for metastasis identified a wide range of metastasis score among cell lines, ranging from 0 to 15617.2 cells (median = 35.4 with interquartile range 9.7 to 75.8, mean ± standard deviation = 867 ± 3397). There was no association between metastatic scores and intrinsic molecular subtypes of the breast cancer cell lines, as defined by Kao *et al* [30] (Fig 1A). Overall, the lung and liver normalized cell counts were highly correlated (Pearson’s product-moment correlation coefficient = 0.6 and P-value <0.01, Fig 1B). However, most cell lines displayed a greater propensity to metastasize to the lung over the liver (cell count median 21 vs. 6, mean of 686 vs. 181), with 12 cell lines with lung > liver, and only 4 cell lines with liver > lung. BT-20, BT-549, and MDA-MB-453 showed the greatest difference in lung compared to liver cell counts. This mimics clinical observation that demonstrates a higher percentage of lung metastasis than liver metastasis in patients with breast cancer [31]. Additional details on the CAM results can be found in Table A in S1 File.

**Table 1:** CAM assay raw lung and liver cell counts and normalized metastasis score.

Cell Line	Subtype	MS	Liver ± SEM	Lung ± SEM
**ACC231**	Lu	4.1	7.4 ± 1.7	0.9 ± 0.2
**ACC302**	BaB	75.8	52.2 ± 5.1	85.4 ± 28.1
**ACC422**	Lu	0	9.8 ± 10.5	10.7 ± 1.9
**ACC440**	BaA	35.4	16.7 ± 3.7	144.2 ± 27.4
**ACC459**	BaB	0	5.1 ± 1	2.8 ± 0.8
**AUA565**	Lu	38.6	18 ± 1.8	64.2 ± 9.7
**BT20**	BaA	1592.6	19.2 ± 7.1	1594 ± 958.4
**BT474**	Lu	12.4	36.6 ± 15.2	15.3 ± 6.3
**BT549**	BaB	326.7	87.5 ± 50.1	249.3 ± 67.7
**CAMA1**	Lu	75.2	70.9 ± 8.9	25.2 ± 7.2
**HCC1806**	NA	9.7	33.2 ± 8	7.2 ± 1.2
**HCC1937**	BaA	0	6.5 ± 2.6	7.1 ± 1.8
**HCC1954**	HER2	127.8	65.5 ± 18.6	77.6 ± 39.7
**HCC38**	BaB	41.1	73.1 ± 10.3	27.2 ± 10.2
**HCC70**	BaA	20.6	19.1 ± 4.3	34.2 ± 17.5
**MCF7**	Lu	124.8	50.2 ± 3.2	110.1 ± 38
**MDAMB231**	BaB	29.9	36.3 ± 6.9	10.5 ± 1.8
**MDAMB361**	HER2	34	33.9 ± 4.4	112.9 ± 51.5
**MDAMB453**	Lu	15617.2	3475 ± 2496.3	12215 ± 5478.2
**T47D**	Lu	0	6.9 ± 1	3.9 ± 1.2
**ZR7530**	HER2	48.8	97.3 ± 38.9	99.9 ± 17.6

Lu = Luminal, BaA = Basal A, BaB = Basal B

### Development of a signature to predict metastatic potential

In order to select genes predictive of the metastatic potential in breast cancer, we compared gene expression data from the breast cancer cell lines to the quantitated metastasis score. Pearson’s correlation was calculated between the expression level of all genes and the quantitated metastasis score from the CAM assay. Genes with a significant correlation and more than a minimum amount of internal variation and expression were selected, as described in the methods. From this, a total of 359 genes were found to be significantly correlated (192 positively correlated, 167 negatively correlated) to the intrinsic metastatic potential as defined in the CAM assay. Heatmap depiction of gene expression across the 20 cell lines with microarray expression data sorted by metastatic potential demonstrates that the expression of these genes correlate well with the metastasis score (Fig 1C). In order to ensure our signature worked in all of our clinical datasets, genes that were not present in all clinical microarrays or genes without Entrez Gene IDs (which were used as the unifying ID) were excluded, leaving 146 genes to be included in M-Sig (Table B in S1 File).

In order to better understand the biological drivers of metastasis, the functions of these 146 genes were investigated using DAVID (Table 2). The top biological processes enriched in the M-Sig gene list were cell migration and immune response, with other nominated concepts including proliferation, inflammation, and hypoxia. In terms of signaling pathways, genes that participate in several known oncogenic signaling pathways such as Jak-Stat, TGF-beta, platelet derived growth factor (PDGF), Wnt, and Ras were also enriched in M-Sig consistent with previously published data [32–36].

**Table 2:** Biological processes and pathways significantly enriched in the M-Sig genes.

Biological Process/Pathway	P-value	Fold Enrich.
Regulation of cell migration	0.0002	5.8
Immune response	0.002	2.5
Regulation of lymphocyte apoptosis	0.01	27.3
Jak-STAT signaling pathway	0.01	4.3
Regulation of cell proliferation	0.01	2.2
Hemopoiesis	0.01	3.7
Nitric oxide mediated signal transduction	0.01	23.4
TGF-beta signaling pathway	0.01	3.8
Immune system development	0.01	3.2
PDGF signaling pathway	0.02	3.2
Response to oxidative stress	0.02	4.0
Wnt signaling pathway	0.03	2.3
Regulation of inflammatory response	0.03	5.7
Leukemia inhibitory factor signaling pathway	0.04	54.5
Negative regulation of apoptosis	0.04	2.5
Cytokine-cytokine receptor interaction	0.05	2.5
BMP signaling pathway	0.06	7.4
Hypoxia response via HIF activation	0.06	7.2
Growth hormone receptor signaling pathway	0.07	27.3
Ras protein signal transduction	0.07	4.2
Mesenchymal cell differentiation	0.08	6.4
Response to host defenses	0.08	24.2
Negative regulation of cell-matrix adhesion	0.09	21.8
Positive regulation of cell cycle	0.09	5.7

### Overview of clinical cohorts

While we demonstrated a strong association between the genes in M-Sig and the intrinsic metastatic potential using an *in vivo* model system, we sought to validate the prognostic power of this signature in human breast cancer patient datasets in which metastatic events and time to event were available. We obtained five publicly available clinical cohorts for signature training and validation. The clinical characteristics of these cohorts have been previously described, but important differences are briefly reviewed below. The cohort from Kao *et al*. was used as the training/cross validation cohort, and consisted of 327 frozen tumor samples from every third patient treated between 1991 and 2004 at the Koo Foundation Sun-Yat-Sen Cancer Center in Taiwan [23]. The median age was 46, median follow-up was 8.1 years, and the patients were heterogeneous in stage, grade, hormone receptor status and treatment modality [23]. The first validation cohort was from Wang *et al*. and was comprised of 286 tumor samples from lymph node-negative patients from the Netherlands who were treated from 1980–95 and who did not receive systemic adjuvant or neoadjuvant therapy [24]. The median age was 52 years, the median follow-up time was 8.4 years for the patients who survived, 97% were T1-2, and the majority (87%) received radiation therapy [24]. The second validation cohort was from van de Vijver *et al*. and comprised 295 consecutive tumor samples from patients from the Netherlands who were treated from 1984–95, and who were diagnosed at age 52 or younger with a tumor less than 5cm in diameter [25]. The median age was 44 and the median follow-up was 7.2 years. The third validation cohort from Hatzis *et al*. was the only prospective dataset, and comprised 508 biopsy samples from patients treated at MD Anderson Cancer Center from 2000–09 (median age 49 years) and who were all treated with neoadjuvant taxane and anthracycline-based chemotherapy regimens [26]. The fourth and most recent validation cohort was from TCGA, and comprised 378 patients (median age 59) with both metastasis free survival and RNAseq data of their tumors [27]. The TCGA cohort had a much shorter median follow-up time of only 1.6 years. These five cohorts represented a broad range of breast cancer patients which include many different clinical and pathologic groups as well as local and systemic treatment modalities. Additional details on these cohorts can be found in Table C in S1 File.

### Training and validation of signature

**Fig 2:**
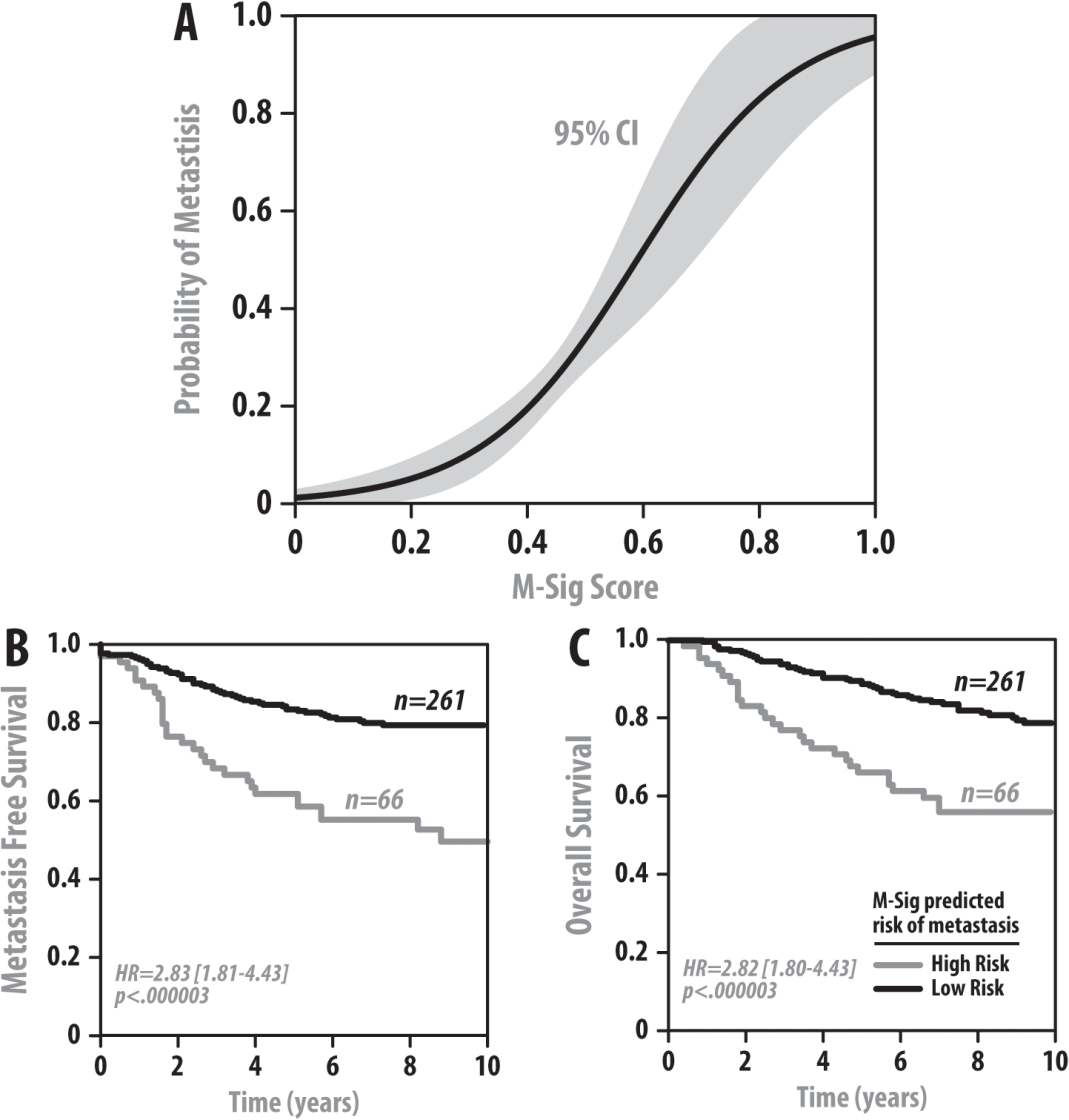
M-Sig Training. (A) M-Sig logistic regression curve demonstrates the sigmoidal relationship between the cross validated OOB M-Sig score (0–1 score representing the M-Sig prediction of metastasis risk, x-axis) and the actual probability of metastasis in the training cohort (y-axis). 95% confidence intervals are also displayed and are fitted tightly around the curve, with an inflection point around 0.5. Kaplan Meier curves depicting metastasis-free survival estimates (B) and overall survival estimates over time (C) demonstrate a significant difference between the groups defined as high versus low risk by M-Sig score. M-Sig was able to significantly distinguish between high vs. low risk for metastasis and OS. Hazard ratios (HR) are shown with 95% confidence intervals.

**Fig 3:**
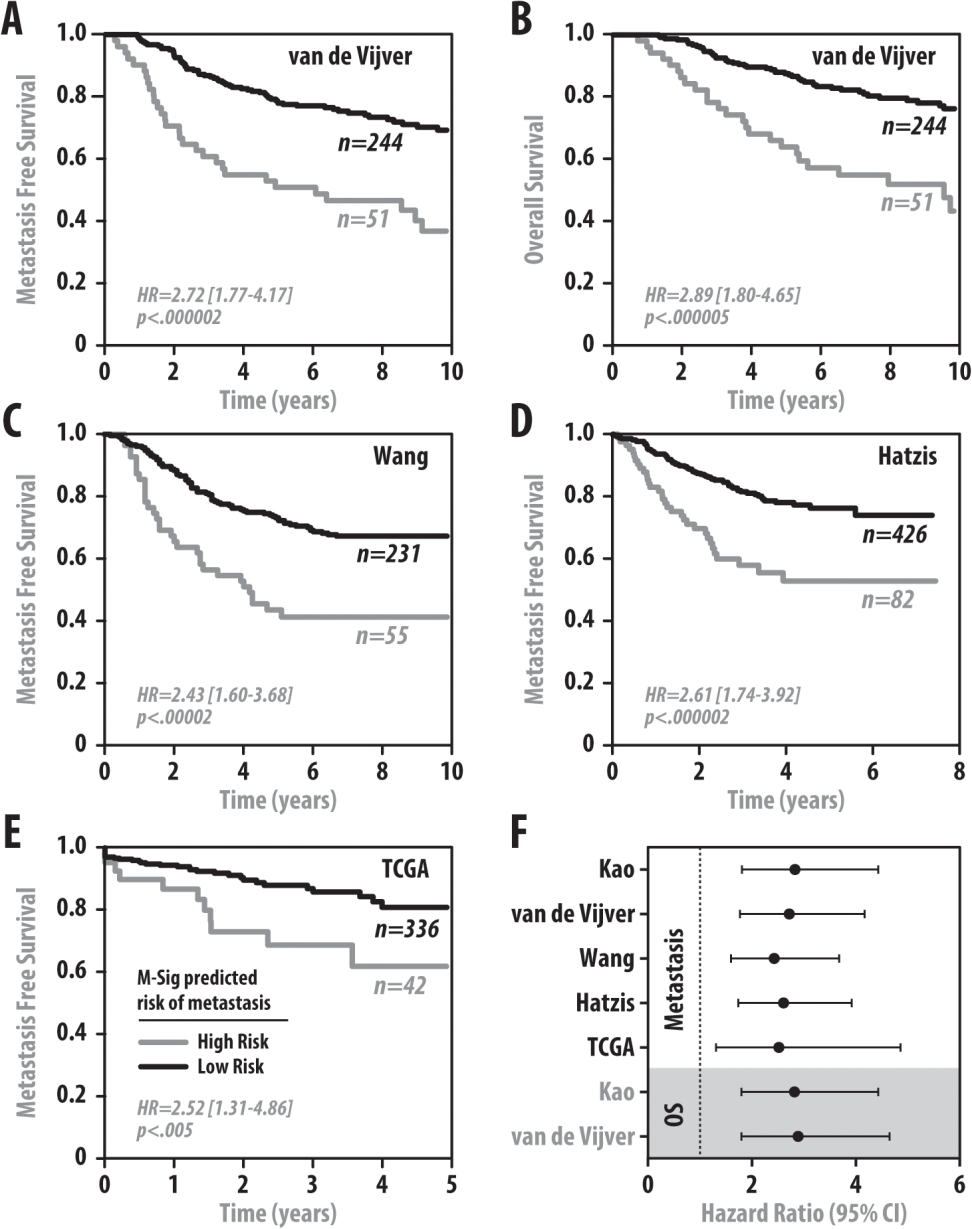
M-Sig Validation. M-Sig is able to risk stratify patients in several independent clinical datasets. Kaplan Meier survival estimate curves for M-Sig predictions for metastasis (A) and OS (B) in the van de Vijver cohort, metastasis in the Wang cohort (C), the Hatzis cohort (D), and the TCGA cohort (E). Hazard ratios (HR) are shown with 95% confidence intervals and a forest plot displays these univariate HRs with confidence intervals for all five cohorts and for both metastasis and overall survival (F). The OOB predictions were used for Kao as in Fig 2.

In order to train the M-Sig classifier in a clinical cohort, we selected the Kao dataset as it was the dataset with the most heterogeneous patient population in terms of clinicopathologic variables and treatments (S2 File). Unsurprisingly, the performance of the final M-Sig model in Kao predicts metastasis perfectly. In order to assess how M-Sig performs in the training set in an unbiased manner, we used the random forest OOB cross validation method. A logistic regression curve was fitted to the OOB predicted M-Sig score (0–1 score which estimates the risk of metastasis) versus the actual events in the training cohort and demonstrates the expected sigmoidal curve with tight confidence intervals suggesting that the relationship between the OOB M-Sig score and the probability of having a metastatic event is tightly correlated (Fig 2A). The inflection point in M-Sig score is approximately 0.5, which is the value we used to divide predicted high versus low risk. We subsequently plot Kaplan-Meier survival estimate curves which compare the OOB M-Sig predicted high versus low risk groups and their actual metastasis rate over time. The OOB M-sig score was able to significantly predict both metastasis (p < 0.001, hazard ratio (HR) = 2.8, 95% confidence interval [1.8–4.4]—Fig 2B) as well as overall survival (OS) (p < 0.001, HR = 2.8 [1.8–4.4]—Fig 2C).We then validated the performance of M-Sig in four additional independent datasets, and show that it is able to stratify metastatic risk in the van de Vijver (p < 0.001, HR = 2.7 [1.8–4.2]), Wang (p < 0.001, HR = 2.4 [1.6–3.7]), Hatzis (p < 0.001, HR = 2.6 [1.7–3.9]), and TCGA (p < 0.005, HR = 2.5 [1.3–4.9]) datasets (Fig 3A, 3C, 3D and 3E). In addition, M-Sig was also able to stratify OS in the van de Vijver dataset (p < 0.001, HR = 2.9 [1.8–4.7]) shown in Fig 3B. OS data was unavailable in Wang and Hatzis, and non-significant in the TCGA dataset which was unsurprising given the low number of death events captured in this clinically immature dataset. These consistent hazard ratios in all 5 cohorts for both metastasis and OS are summarized in Fig 3F. Clinical and pathological variable data were available in the van de Vijver, Hatzis, and TCGA datasets as well, and M-Sig was significantly prognostic for metastasis in van de Vijver (p < 0.001, HR = 2.3 [1.5–3.6]), Hatzis (p < 0.001, HR = 2.2 [1.4–3.5]), and TCGA (p < 0.01, HR = 6.7 [1.9–24]) and OS in van de Vijver (p < 0.05, HR = 1.9 [1.2–3.2]) on multivariable analysis (MVA) in Table 3. These results demonstrate that M-Sig can significantly stratify metastatic potential of breast cancers in clinical cohorts, and is the top predictor on MVA with stronger hazard ratios than all other clinical-pathologic variables on MVA. In the van de Vijver cohort, other significant clinical variables besides M-Sig include age (HR = 0.95 [0.92–0.98], p < 0.001), grade (HR = 1.5 [1.1–2.1], p<0.001), and node status (HR = 1.1 [1.0–1.2], p<0.05) on MVA for metastasis. In the Hatzis cohort, M-Sig was once again able to significantly predict metastasis with the largest HR of 2.2 on MVA with the other significant variables being node status (HR = 1.5 [1.2–1.9], p<0.00001), tumor stage (HR = 1.3 [1.0–1.7], p<0.05), and ER status (HR = 0.5 [0.3–0.9], p<0.05), Finally, in TCGA which was an RNAseq cohort, M-Sig was the only significant predictor of metastasis on MVA.

**Table 3:** Univariate and multivariate analysis of M-Sig.

		Univariate		Multivariate		
Cohort	Covariates	P-value	HR	P-value		HR
Van de Vijver (Metastasis)	Diameter (mm)	0.00046	1.42 [1.17–1.74]	0.055		1.24 [1–1.54]
	LN (# positive)	0.021	1.09 [1.01–1.18]	0.030		1.1 [1.01–1.2]
	Mastectomy vs. BCT	0.10	1.39 [0.94–2.05]	0.48		1.17 [0.76–1.79]
	ER	0.0049	0.54 [0.36–0.83]	0.93		1.02 [0.64–1.63]
	Grade	<0.0001	1.97 [1.49–2.6]	0.0046		1.54 [1.14–2.08]
	Age (yr)	0.00024	0.94 [0.91–0.97]	0.0025		0.95 [0.92–0.98]
	Chemo	0.25	0.79 [0.52–1.19]	0.051		0.61 [0.37–1]
	Hormonal	0.14	0.58 [0.28–1.19]	0.36		0.7 [0.33–1.49]
	M-Sig (high/low)	<0.0001	2.72 [1.77–4.17]	0.00038		2.29 [1.45–3.62]
Van de Vijver (OS)	Diameter (mm)	0.0011	1.45 [1.16–1.8]	0.20		1.18 [0.92–1.51]
	LN (# positive)	0.17	1.07 [0.97–1.17]	0.16		1.08 [0.97–1.19]
	Mastectomy vs. BCT	0.41	1.2 [0.77–1.87]	0.58		1.15 [0.71–1.86]
	ER	<0.0001	0.3 [0.19–0.48]	0.016		0.55 [0.34–0.89]
	Grade	<0.0001	2.69 [1.89–3.84]	0.00078		1.92 [1.31–2.8]
	Age (yr)	0.0041	0.94 [0.91–0.98]	0.038		0.96 [0.92–1]
	Chemo	0.33	0.79 [0.49–1.26]	0.31		0.74 [0.42–1.32]
	Hormonal	0.24	0.61 [0.26–1.4]	0.59		0.79 [0.33–1.87]
	M-Sig (high/low)	<0.0001	2.89 [1.8–4.65]	0.011		1.94 [1.16–3.23]
Hatzis (Metastasis)	Age (yr)	0.86	1 [0.98–1.02]	0.50	0.99 [0.97–1.01]	
	ER	<0.0001	0.34 [0.23–0.51]	0.019	0.52 [0.3–0.9]	
	PR	<0.0001	0.38 [0.25–0.57]	0.54	0.84 [0.48–1.47]	
	HER2	0.43	1.76 [0.43–7.13]	0.63	1.42 [0.34–5.89]	
	T-stage	0.00031	1.51 [1.21–1.88]	0.026	1.31 [1.03–1.67]	
	N-stage	<0.0001	1.66 [1.38–2.01]	<0.0001	1.52 [1.23–1.87]	
	Grade	0.0056	1.65 [1.16–2.35]	0.86	1.03 [0.7–1.53]	
	M-Sig (high/low)	<0.0001	2.61 [1.74–3.92]	0.00066	2.22 [1.4–3.51]	
TCGA (Metastasis)	Age	0.87	1 [0.98–1.02]	0.50	0.99 [0.96–1.02]	
	N-stage	0.61	0.92 [0.68–1.26]	0.44	0.85 [0.55–1.29]	
	T-stage	0.65	1.1 [0.74–1.63]	0.79	1.08 [0.61–1.92]	
	Close vs. Negative Margins	0.52	0.52 [0.07–3.81]	0.21	0.24 [0.03–2.24]	
	Positive vs. NegativeMargins	0.90	1.07 [0.38–2.99]	0.83	0.84 [0.19–3.84]	
	Mastectomy vs. BCT	0.24	0.68 [0.35–1.3]	0.63	0.83 [0.4–1.75]	
	Basal vs. Normal	0.63	1.44 [0.32–6.44]	0.82	1.29 [0.15–10.88]	
	Her2 vs. Normal	0.76	0.76 [0.13–4.55]	0.51	0.43 [0.04–5.18]	
	LumA vs. Normal	0.61	0.69 [0.16–2.95]	0.33	2.86 [0.34–24.22]	
	LumB vs. Normal	0.98	0.98 [0.22–4.44]	0.35	2.78 [0.32–23.77]	
	M-Sig (high/low)	0.0058	2.52 [1.31–4.86]	0.0032	6.73 [1.9–23.9]	

Hazard ratios are shown with 95% confidence intervals

## Discussion

Metastasis is a complex biological process in which a cancer cell must successfully complete a series of steps in order to successfully grow in a distant anatomic site. This process is responsible for >90% of mortality in cancer [8]. While surgery and radiation have the potential to cure localized disease, metastatic cancer is considered incurable by conventional therapies [8]. Therefore, understanding and predicting which tumors have the potential to metastasize is critical in guiding therapy and developing new treatments.

To address this important clinical need, we have developed M-Sig, the first gene expression signature that predicts intrinsic metastatic potential based on a large scale *in vivo* metastasis assay to assess the intrinsic metastatic potential of breast cancer cell lines. In human samples, M-Sig robustly differentiated between patients at higher vs. lower risk of metastasis, on cross validation in the training cohort, as well as in four additional independent validation cohorts. Incredibly, M-Sig achieved a consistent hazard ratio of approximately 2.5–3, with highly significant p-values in all five clinical cohorts despite completely different sample procurement, processing, and gene expression platforms including RNAseq. This represents the first truly platform-independent predictor of metastasis in breast cancer.

In breast cancer, the use of gene expression signatures to predict prognosis have become a part of standard clinical practice. However, existing signatures such as MammaPrint and Oncotype Dx are not “pure” metastasis signatures due to how they were derived. MammaPrint is a 70 gene prognostic signature derived by selecting the genes that best correlated with distant metastasis within 5 years in a clinical cohort of 78 node-negative patients [5]. Oncotype Dx is a 21 gene classifier developed by selecting 250 genes based on manual curation of the literature for breast cancer biomarkers [6]. This list was narrowed to 21 genes based on their prognostic ability in three independent clinical cohorts [6]. Oncotype Dx has also been validated as predictive for response to chemotherapy in ER-positive, node-negative breast cancer [37]. Since both signatures selected their genes based on biomarker potential in clinical cohorts, they include some combination of the intrinsic metastatic potential of the tumor and its resistance to conventional treatments. Prosigna has also developed a prognostic signature based on the PAM50 molecular subtypes of breast cancer [7]. Though the PAM50 subtypes are prognostic, they were not selected to predict intrinsic metastatic potential either [3].

Therefore, with existing molecular signatures it is possible to predict whether a given patient will do poorly but it is not yet possible to determine if poor prognosis is due to high intrinsic metastatic potential of the tumor, or if the tumor is radio, chemo, or endocrine therapy-resistant, or even if has characteristics which make it difficult to completely surgically excise. M-Sig may represent a truer measure of metastatic potential independent of treatment effect as it was derived primarily from an *in vivo* model system. It should be noted that M-Sig does not represent a completely “pure” metastasis signature as it was trained in a clinical cohort. Though it would have been experimentally ideal for the clinical outcomes to be independent of treatment, obviously datasets with untreated breast cancer do not exist, nor would it be ethical to collect such data. However, the relatively uniform performance of our signature between cohorts with different treatments lends evidence that M-Sig is actually predicting independent of treatment.

M-Sig could be used in combination with specific treatment response signatures in order to personalize treatment. One can envision a situation where the tumor is predicted to not benefit from standard chemotherapy (low Oncotype Dx score), but has a high metastatic potential (as assessed by M-Sig), in which case the optimal treatment may involve intensified local and/or systemic therapy. Another potential use for M-Sig is in the research setting, where it could be used as an inexpensive and rapid way to measure the effect of a particular experimental condition on all the steps in metastasis. The platform-independent nature of M-Sig is particularly advantageous, as it can be applied to any dataset without modification.

In addition to providing a prognostic tool that helps identify patients with breast cancers at high risk for systemic progression, M-sig also sheds insight into the biological processes underlying metastatic spread. The M-Sig genes identified in this study are involved in both general processes critical to metastasis such as cellular adhesion, migration, proliferation, cell cycle regulation, cytokine signaling, and immune cell evasion [38], as well as canonical oncogenic pathways such as the TGF-beta [33], Jak-Stat [32], PDGF [34], Wnt [35], and Ras [36] signaling pathways. In particular, the enrichment of genes associated with TGF-beta signaling in M-Sig is of particular interest. Several key genes in the TGF-beta pathway are integral to M-Sig and are positively correlated with metastatic potential including Activin R1 (ACVRL1) and SMAD6. Additionally, the T47D cell line, known to be deficient in TGF-beta receptors I-II [39], demonstrated no metastatic potential in the CAM assays. Strikingly, TGF-beta receptor 3, an inhibitor of TGF-beta signaling is negatively correlated with metastatic potential, consistent with the literature [40]. Our study also identifies immune system regulation as 8 of the 16 top biological processes associated with metastatic potential. The role of the immune system in regulating metastatic potential and patient outcomes has become an area of active investigation in recent years, with many groups identifying immunophenotypes associated with favorable and unfavorable outcomes in patients with breast cancer [41–44]. Our study adds to the growing body of literature that immunoregulatory genes may be expressed by the epithelial cancer cells to affect the local immune response. Indeed, this has been reported in previous studies and suggests there is a more complex interplay between the circulating immune monitoring cells and tumor cells seeking to avoid detection [44, 45].

While our findings represent the largest *in vivo* study to date quantifying the metastatic potential in human breast cancer, there are certain limitations. The CAM model system is not a perfect analog of human metastasis, and metastasis to chick organs may not be completely representative of disease progression and metastasis in humans. The chick embryos used are also not fully immunocompetent and therefore cannot fully model the role of the immune system in metastasis. Additionally, this system does not easily allow for the assessment of all common sites of breast cancer metastasis, including the brain and bone. Finally, the timescale of the CAM model system is much shorter than the natural history in human disease.

An additional potential drawback of our model system is that, using the CAM assay, we did not find a correlation between molecular subtype and metastatic risk, which differs substantially from clinical findings. This result likely stems from the fact that cell lines do not represent the complete spectrum of breast cancer. Rather, cell lines tend to be from typically aggressive tumors and represent a subset of cancer cells which have been immortalized and are able to grow independent of the interactions present in the tumor microenvironment. Additionally, the relatively small number of cell lines in each group and the wide range of metastasis scores within each subtype limits the ability to identify a statistically significant difference in subtype-dependent metastatic potential. Interestingly, the MCF7 cell line, which is considered to be of low metastatic potential based on nude mice and Boyden chamber assays [10, 46], demonstrates significant metastatic potential in our data. Additionally, MDA-MB-453, which has been categorized as non-metastatic in prior reports [10], was the most metastatic cell line to both the lung and liver. Since both these cell lines was derived from metastatic pleural and pericardial effusions respectively, our data is more consistent with what would be expected biologically, demonstrating that the CAM assay may assess metastatic potential not detectable with more conventionally used metastatic assays.

Another limitation in our study is the technical and biological differences between the publicly available clinical and microarray datasets. Therefore, unsurprisingly, the clinical signatures derived in each of the publicly available or similar cohorts tend to perform better than M-Sig (HR = 5.7 for the Wang signature, HR = 5.3 for the Hatzis signature, HR = 5.1 for Mammoprint in the Van de Vijver cohort). However, the performance of M-Sig across all available datasets is highly significant and consistent, which is a remarkable finding considering the vast amount of heterogeneity in and between the clinical datasets. In fact, because our signature focuses on predicting only metastasis without treatment effect, we would have expected it to perform poorer in treatment confounded clinical cohorts.

In conclusion, we present the first high-throughput *in vivo* screen to characterize the intrinsic metastatic potential in breast cancer cell lines. We used these data to develop M-Sig, the first platform-independent gene signature designed to predict intrinsic metastatic potential, and validate in five clinical cohorts with nearly 1800 patients. This signature has a myriad of potential clinical and research applications and represents a significant addition to the compendia of available prognostic signatures in breast cancer.

## Supporting Information

S1 File Supplemental Tables A-C. *Table A in S1 File*. *CAM assay results*: This table contains the raw CAM assay results for each cell line, as well as the normalized lung, liver, and metastasis scores. *Table B in S1 File*. *M-sig gene list*: This table contains Spearman’s Rho and p-value for correlation with the CAM metastatic potential in the cell line microarrays for each gene. The expression percentiles indicate the 25th, 50th (median), and 75th percentiles of Log2 transformed gene expression in the cell line microarray data to indicate the level as well as the range of gene expression for each gene. *Table C in S1 File*. *Database demographics*: This table contains available demographic and clinical data on each cohort used in the study.
(XLSX)

S2 File M-Sig model. The M-Sig model is a computational model which is created using the R ‘randomforst’ package. The actual model consists of 100,000 individual decision trees with different combinations and configurations of genes which were trained on random subsets of the training dataset as described in the manuscript. This collection is organized in S2 File, which contains M-Sig as an R ‘randomforest’ object. This allows M-Sig to be loaded into R using the ‘load’ command, and provides a computational interface with which to generate M-Sig scores on new datasets. The input gene expression data should be formatted as described in the manuscript, i.e. gene expression should be log transformed, median centered and scaled. The columns must represent individual genes, and column names for the input data must be the Entrez gene ID prefixed by an X (i.e. X3924). The rows represent individual samples, and the row names for the input data can be any unique identifier. Expression data is required for all genes for M-Sig to work properly. Once the object is loaded, predictions can be generated with the ‘predict’ function as described in the R ‘randomforest’ documentation, which will generate an M-Sig score for every sample.
(ZIP)
